# Identification of oxyresveratrol, a food-as-medicine substrate, as a novel EPI that targets the RND efflux pumps of *Serratia surfactantfaciens* sp. nov. YD25^T^

**DOI:** 10.1128/spectrum.02185-25

**Published:** 2026-04-22

**Authors:** Fuqiao Liu, Xiaoyu Wei, Yaoyu Lou, Yurong Wang, Danlu Zhang, Bing Cao, Chun Su, Yan Sun, Zhi Li

**Affiliations:** 1 College of Life Sciences, Shaanxi Normal University 12401, Xi’an, People's Republic of China; JMI Laboratories, North Liberty, Iowa, USA

**Keywords:** efflux pump inhibitor, food-as-medicine substrate, oxyresveratrol, RND, Rob, *Serratia*

## Abstract

*Serratia marcescens* is an opportunistic pathogen with increasing drug resistance. *Serratia surfactantfaciens* sp. nov. YD25^T^, a novel strain with multidrug resistance, shares similarities with *S. marcescens*. This study investigated the resistance mechanisms of YD25, with a focus on efflux pumps and their regulators. Bioinformatics analysis revealed numerous resistance genes in YD25, including five RND-type efflux pumps, SdeAB, SdeCDE, SdeGH, SdePQ-OmsA, and SdeXY. Oxyresveratrol, evernic acid, and morin were identified as efflux pump inhibitors, with oxyresveratrol showing the strongest effect targeting SdeXY and sensitizing YD25 to seven antibiotics. Among the RND pumps, SdeXY and SdeCDE are constitutively expressed and are regulated negatively by AcrR and positively by BaeSR, respectively. YD25 harbors four global regulators, including RamA, SoxS, Rob, and a novel Roblike protein. The deletion of these genes downregulated SdeXY and SdeCDE, increasing antibiotic susceptibility and confirming their role as positive regulators. Rob and Roblike exhibited functional overlap. This study elucidates the RND efflux pumps and their regulators in YD25, advancing the understanding of *Serratia* resistance mechanisms. Oxyresveratrol, a newly identified SdeXY inhibitor, offers potential for combination therapies against drug-resistant *Serratia*.

**IMPORTANCE:**

This study reveals novel insights into *Serratia surfactantfaciens* YD25’s multidrug resistance, identifying RND efflux pumps (e.g., SdeXY and SdeCDE) as key drivers. The key discoveries include the following: (i) oxyresveratrol, a natural compound functioning as an SdeXY efflux pump inhibitor, effectively reverses bacterial resistance, thereby restoring the clinical utility of antibiotics that had been rendered nearly obsolete by drug resistance, and (ii) YD25 uniquely possesses two Rob regulators (Rob and Roblike). Despite their different origins, both positively regulate RND pump function—a finding reported for the first time in bacteria. These findings highlight the potential of natural efflux pump inhibitors against *Serratia* infections while uncovering an unusual regulatory redundancy in bacterial resistance.

## INTRODUCTION

*Serratia marcescens*, a member of the “ESCPM” group, is demonstrating increasing multidrug resistance among clinical isolates (1). Bacterial resistance encompasses both acquired and intrinsic mechanisms, with the latter contributing significantly to resistance through altered membrane permeability, target site mutations, drug degradation, and efflux pump activity (2). Among these mechanisms, efflux pumps are particularly notable due to their broad distribution and substrate versatility (3). The RND superfamily, one of the seven known efflux pump families, is distinguished by its ability to reduce intracellular concentrations of diverse antimicrobials, making it a key resistance mechanism in many gram-negative pathogens (4).

The *Serratia* genus possesses multiple families of efflux pumps. A study analyzing the whole genomes of 32 *Serratia* strains identified 74 efflux pump-encoding genes belonging to the MATE, SMR, MFS, ABC, and RND families. Among these, the RND family was found to be the most abundant, followed by the MFS and ABC families, while the recently discovered PACE pump and AbgT transporter protein were not found in *Serratia* (5). As the most prevalent efflux pump family in gram-negative bacteria, RND is the key player in multidrug resistance (6). The RND efflux pump is composed of three complexes that can transport substrates across the inner and outer membranes of gram-negative bacteria. Therefore, the RND efflux pump not only directly pumps intracellular substrates out of the cell but also transports substrates from families such as SMR, MFS, and MATE to the periplasmic space out of the cell (7). The first reported RND efflux pump in *S. marcescens* was SdeXY, followed by SdeAB and SdeCDE (8–10). In the *S. marcescens* Db10 strain, in addition to the reported SdeXY, SdeAB, and SdeCDE pumps, members of the RND family, such as SdeGH, SdeIJ, SdeNO, and SdePQ-OmsA, have been identified (11). Other studies have also revealed the presence of orthologous families of efflux pumps in different *S*. *marcescens* strains. For instance, *S. marcescens* NUSM8903 features SsmE and SmfY, which are homologous to SMR family EmrE (*Escherichia coli*) and MFS family QacA (*Staphylococcus aureus*), respectively (12, 13). *S. marcescens* NUSM8906 harbors SmdAB, an ABC superfamily pump homolog of *E. coli* MdlAB (14), and SdeXY, an RND family pump highly homologous to *E. coli* AcrAB (8). ABC and MFS transporters are both prevalent in gram-positive bacteria; however, in contrast to the limited role of ABC pumps in multidrug resistance, the MFS family is closely linked to this function.

The regulatory patterns of RND efflux pumps are highly similar across different bacterial species. The production of efflux pumps is strictly controlled, with the most direct regulation coming from local regulators, most of which belong to the Tet family. For example, in *S. marcescens* UOC-67, the production of the SdeAB efflux pump is tightly regulated by two adjacent genes: the *sdeS* gene, located downstream of the *sdeAB* operon in a tail-to-tail orientation and encoding a repressor that likely inhibits *sdeAB* transcription by binding to its promoter, and the upstream *sdeR* gene, which encodes an activator that promotes its expression (15, 16). In addition to local regulators, many global transcriptional regulators affect the expression of efflux pump genes. Key examples include MarA, SoxS, Rob, and RamA, which predominantly belong to the AraC/XylS family. *S. marcescens* RM66262 is a clinical isolate in which, after *ramA* was deleted, the expression of the efflux pump gene *acrAB-tolC* was downregulated, whereas when *ramA* was overexpressed, the sensitivity of the strain to tetracycline, nalidixic acid, and chloramphenicol was reduced (17).

Efflux pump inhibitors (EPIs) have the potential to restore the antibacterial effects of antibiotics, either by directly binding to efflux pump proteins or by cutting off the energy supply to the efflux pump (18). Many compounds may serve as EPIs; for example, discovered EPIs in *E. coli* include indoles, arylpiperazines, hydantoins, catechols, geraniol derivatives, quinolines, and quinazolines (19). “Medicine and Food Homology” is a traditional Chinese medicine theory that refers to substances that can be used both as food and medicine. In addition to their nutritional value, “food as medicine” products also have preventive and therapeutic effects, as well as other health benefits. Medicines inevitably have certain side effects, and food itself is safer; thus, the use of food as medicine has many advantages. By the end of 2023, the Chinese State Council’s health administrative department had announced a total of 102 “food as medicine” substances, which are high-quality resources for exploring novel and safe EPIs.

In our previous study, we identified a novel *Serratia* species, *Serratia surfactantfaciens* sp. nov. YD25 (YD25), which is highly resistant to multiple antibiotics (20). Genomic sequencing revealed that this strain lacks plasmids and carries resistance genes on its chromosome, confirming that its resistance is primarily intrinsic. This study focused on intrinsic resistance mechanisms, particularly the role of RND efflux pumps in the multidrug resistance of YD25. Additionally, we screened for safe and effective EPIs and investigated their mechanisms. As YD25 is phylogenetically closely related to *S. marcescens*, this study provides insights into both the resistance of this new species and the multidrug resistance of *S. marcescens*, aiding in the development of clinical drug use strategies.

## RESULTS

### The resistance of YD25 to antibiotics

The resistance of YD25 was detected, and the results revealed that YD25 had high minimum inhibitory concentrations (MICs) for all the tested antibiotics (Table 1). The EUCAST data revealed that different strains of *S. marcescens* from various sources presented MICs for each antibiotic within a certain range. For example, among 4,109 strains of *S. marcescens*, 2,956 strains had an MIC of 32 μg/mL for ampicillin, and 151 strains had an MIC of 512 μg/mL. With an ampicillin MIC of 512 μg/mL, YD25 exhibits pronounced resistance relative to the MIC distribution of 4,109 *S. marcescens* strains. Similarly, YD25 showed a certain degree of resistance to most of the tested antibiotics. Thus, YD25 represents a notable, severe MDR strain of *Serratia*.

**TABLE 1:** Sensitivity of YD25 to different antibiotics^*a*^

Types of antibiotics		YD25	MIC distributions for *S. marcescens*, update to 2025-07-10																
		MIC (μg/mL)	0.008	0.016	0.03	0.06	0.125	0.25	0.5	1	2	4	8	16	32	64	128	256	512
Penicillins	Ampicillin	512	0	0	0	0	0	**1**	**2**	**1**	**10**	**58**	**151**	**358**	**2,956**	**184**	**126**	**111**	**151**
Cephalosporins	Cefazolin	1,024																	
	Cefotaxime	1,024	0	0	**1**	**8**	**19**	**36**	**47**	**39**	**13**	**7**	**5**	**6**	**9**	**10**	0	4	0
	Cefalexin	256																	
	Cefazedone	16																	
Aminoglycosides	Kanamycin	32	0	0	0	0	0	0	0	0	**26**	**32**	**8**	**4**	**2**	**1**	0	2	0
	Gentamicin	8	0	0	0	**2**	**17**	**124**	**819**	**403**	**56**	**22**	**32**	**46**	**54**	**19**	**8**	**50**	**156**
	Streptomycin	8																	
Tetracyclines	Tetracycline	256	0	0	0	0	0	0	**1**	**1**	**4**	**25**	**12**	**12**	**20**	0	0	0	0
	Doxycycline	32	0	0	0	0	0	**1**	**1**	**4**	**20**	**48**	**50**	**38**	**11**	**1**	0	0	0
Macrolides	Erythromycin	128																	
Fluoroquinolones	Nalidixic acid	8																	
	Ciprofloxacin	8	**6**	**8**	**67**	**211**	**298**	**57**	**52**	**85**	**68**	**49**	**25**	**6**	**8**	**7**	**2**	0	0
	Norfloxacin	16																	
Miscellaneous agents	Chloramphenicol	128																	
	Spectinomycin	16																	

^*a*^ The MIC distributions for *S. marcescens* were cited from EUCAST (https://www.eucast.org/). Taking ampicillin as an example, there is one strain of *S. marcescens* with an MIC of 0.25 μg/mL, two strains with an MIC of 0.5 μg/mL, and so on. The highest number of strains, 2,956, occurs at an MIC of 32 μg/mL. The data with the highest quantity are highlighted in bold and underlined. Missing data indicate that EUCAST did not provide the information.

YD25 contains various resistance genes in its genome, which can be divided into two categories according to Resistance Gene Identifier (RGI) prediction. The genes belonging to the first category function by inactivating antibiotics, changing the target of antibiotics, and other means, such as srt-3, aac(6′)-Ic, and arnT (Table S1). The second category is related to efflux pumps. Genomic annotation of YD25 confirmed the predicted presence of genes encoding efflux transporter proteins, identifying a total of 41 genes spanning the RND, MFS, SMR, MATE, and ABC families (Table S2). Combined with the absence of plasmids in YD25, the MDR phenotype of YD25 can be attributed to its chromosomally encoded resistance genes.

### Natural compounds that act as efflux pump inhibitors affect YD25 resistance

The MICs of the compounds from the Food as Medicine Compound Library were tested against YD25, and the growth curve of YD25 under the treatment of sub-MIC (1/2× MIC and 1/5× MIC) candidate compounds was measured in our previous experiments (Selected test results are presented in Fig. S1). Finally, similar to other reports, a sub-inhibitory concentration (1/2× MIC, 1/5× MIC, 1/10× MIC, and 1/20× MIC) that showed no significant effect on YD25 was used for the EPI screening assay (21).

**Fig 1:**
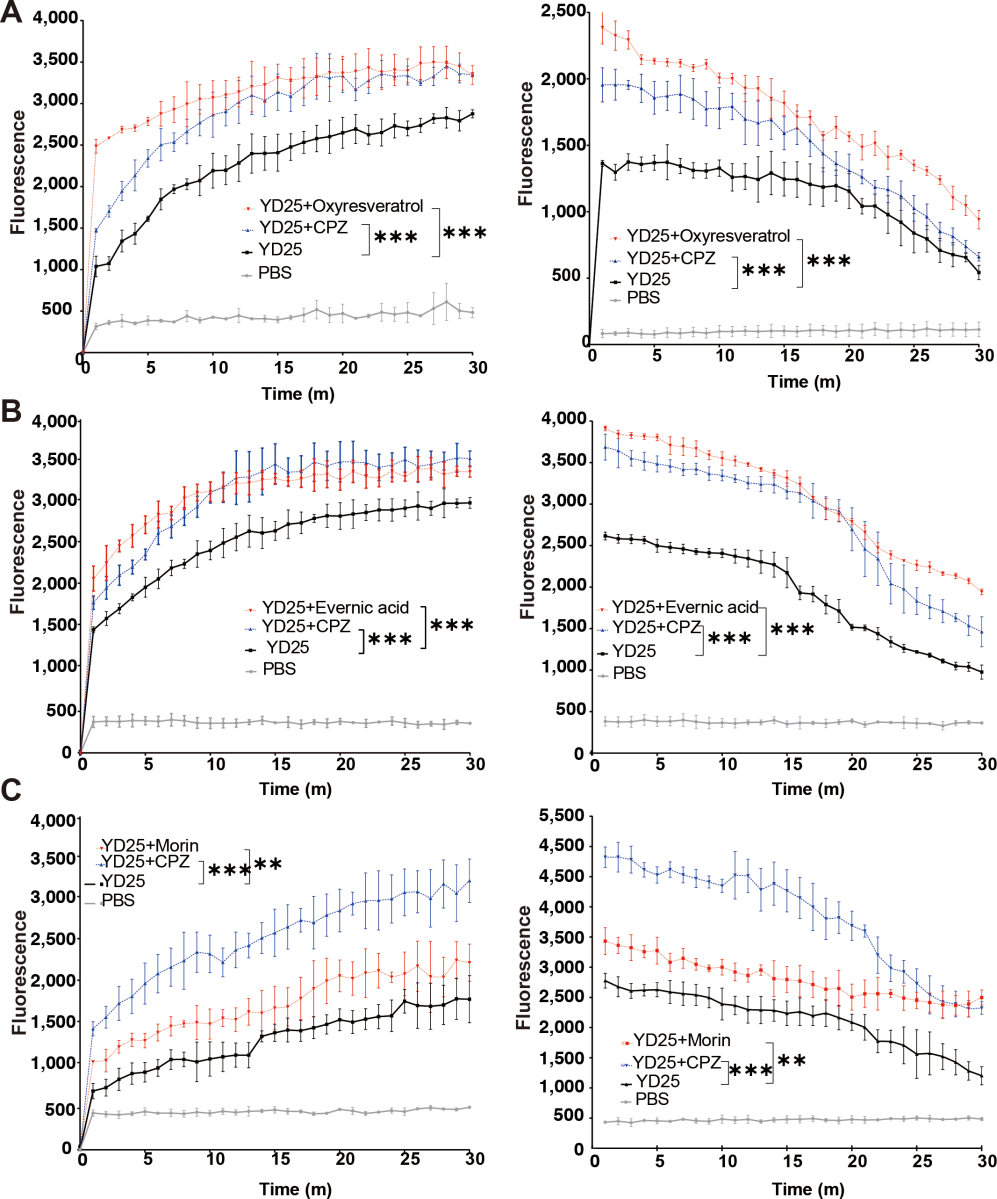
Hoechst accumulation and efflux assay. Representative results show the maximum efflux inhibition achieved by three compounds at their optimal active concentrations: oxyresveratrol at 1/20× MIC (**A**), evernic acid at 1/2× MIC (**B**), and morin 1/2× MIC (**C**). In each graph, the red line represents the compound-treated group, the blue line represents the CPZ-treated positive control group, the black line represents untreated YD25, and the gray line represents the blank PBS solution, with the x-axis denoting the treatment time and the y-axis denoting the fluorescence intensity. *P < 0.01 and ***P < 0.001.

Oxyresveratrol at a concentration of 1/20× MIC demonstrated the strongest inhibitory effect on the YD25 efflux pump (Fig. 1A), outperforming not only oxyresveratrol at 1/2× MIC (Fig. S1) but also numerous other compounds tested at various concentrations. In the dye accumulation experiment, the fluorescence value of the experimental group with oxyresveratrol remained high, even higher than that of the CPZ-positive control group, indicating that oxyresveratrol prevented the efflux of fluorescent dye taken up by cells. In the dye efflux experiment, the oxyresveratrol group presented the slightest decrease in fluorescence value after the cells had taken up enough fluorescent dye, which was even slower than that of the CPZ group. Therefore, oxyresveratrol is an efflux pump inhibitor for YD25. Additionally, 1/2 MIC× evernic acid had an inhibitory effect on the efflux pump similar to that of the positive control CPZ, while the effect of 1/2 MIC× morin was not as strong as that of CPZ but was better than that of the negative control (Fig. 1B and C). Therefore, evernic acid and morin are also efflux pump inhibitors for YD25.

Seven antibiotics were selected for combination therapy with the above three natural efflux pump inhibitors (Table 2). The results showed that after the combination of oxyresveratrol and antibiotics, the sensitivity of YD25 to the seven antibiotics improved. For ampicillin, erythromycin, and tetracycline, the sensitivity increased by 2–4 times, whereas the sensitivity of the other four antibiotics increased by more than 16 times. After the combination of evernic acid and antibiotics, the sensitivity of YD25 to cefotaxime, kanamycin, and chloramphenicol increased by 32, 8, and 4 times, respectively, and the sensitivity to the other four antibiotics increased by two times. For morin and antibiotics, the sensitivity of YD25 to tetracycline did not change, but the sensitivity to the other six antibiotics increased by 2–60 times.

**TABLE 2:** MIC of YD25 against the combination of antibiotics and efflux pump inhibitors^*a*^

Antibiotics		MIC μg/mL (fold)				
			CPZ	Oxyresveratrol	Evernic acid	Morin
Penicillins	Ampicillin	512	512 (1)	256 (−2)	256 (−2)	128 (−4)
Cephalosporins	Cefotaxime	1,024	8 (−128)	32 (−32)	32 (−32)	16 (−64)
Aminoglycosides	Kanamycin	32	4 (−8)	2 (−16)	4 (−8)	2 (−16)
Tetracyclines	Tetracycline	256	128 (−2)	64 (−4)	128 (−2)	256 (1)
Macrolides	Erythromycin	128	64 (−2)	64 (−2)	64 (−2)	64 (−2)
Fluoroquinolones	Nalidixic acid	8	1 (−8)	0.5 (−16)	4 (−2)	2 (−4)
Miscellaneous agents	Chloramphenicol	128	16 (−8)	8 (−16)	32 (−4)	16 (−8)

^*a*^ All seven antibiotics were individually combined with each of the three efflux pump inhibitors at specified concentrations: oxyresveratrol at 1/20× MIC, and evernic acid and morin at 1/2× MIC. CPZ was used as the positive control at a concentration of 1/2× MIC. In the fold-change values, a negative number indicates a decreased MIC relative to the control group, suggesting increased bacterial susceptibility to the stimulants; whereas a positive number indicates enhanced bacterial tolerance to the stimulants.

The checkerboard assay revealed that the MIC values of all seven tested antibiotics decreased when combined with oxyresveratrol. Except for ampicillin and erythromycin, which exhibited an additive effect, the other five antibiotics demonstrated synergistic effects with oxyresveratrol (Table 3). Among these, cefotaxime and chloramphenicol showed the lowest FICI values, reaching 0.14.

**TABLE 3:** MICs of different antibiotics in combination with oxyresveratrol^*a*^

Antibiotics		MIC alone (μg/mL)	MIC in combination (μg/mL)		FICI
			Antibiotics	Oxyresveratrol	
Penicillins	Ampicillin	512	256	425	1.00
Cephalosporins	Cefotaxime	1,024	16	106.5	0.14
Aminoglycosides	Kanamycin	32	4	53.1	0.19
Tetracyclines	Tetracycline	256	16	106.5	0.19
Macrolides	Erythromycin	128	64	425	1.00
Fluoroquinolones	Nalidixic acid	8	0.5	212.5	0.31
Miscellaneous agents	Chloramphenicol	128	2	106.5	0.14

^*a*^ FICI categories: ≤0.5, synergistic; >0.5 to ≤1, additive; >1 to <4, no interaction; ≥4, antagonism.

Molecular docking provided further evidence that oxyresveratrol, evernic acid, and morin may directly bind to efflux pumps. Oxyresveratrol binds to the SdeY protein of the SdeXY efflux pump with a binding energy of −7.39 kcal/mol, primarily stabilized by van der Waals forces and conventional hydrogen bonds (Fig. S2A). Morin binds to SdeQ with a binding energy of −8.25 kcal/mol, primarily stabilized by van der Waals forces, conventional hydrogen bonds, and carbon-hydrogen bonds (Fig. S2B). Evernic acid binds to SmdB with a binding energy of −6.19 kcal/mol, stabilized by multiple interaction forces (Fig. S2C). An extensive interaction network with multiple amino acid residues was observed for each compound, as detailed in Fig. S1D. Integrating bioinformatic analysis results with experimental findings allows for a reasonable hypothesis to be proposed: oxyresveratrol, evernic acid, and morin serve as efflux pump inhibitors in the YD25 strain by blocking the transport of antibiotics to increase sensitivity in YD25.

### Expression and efflux activity of RND efflux pumps in YD25

**Fig 2:**
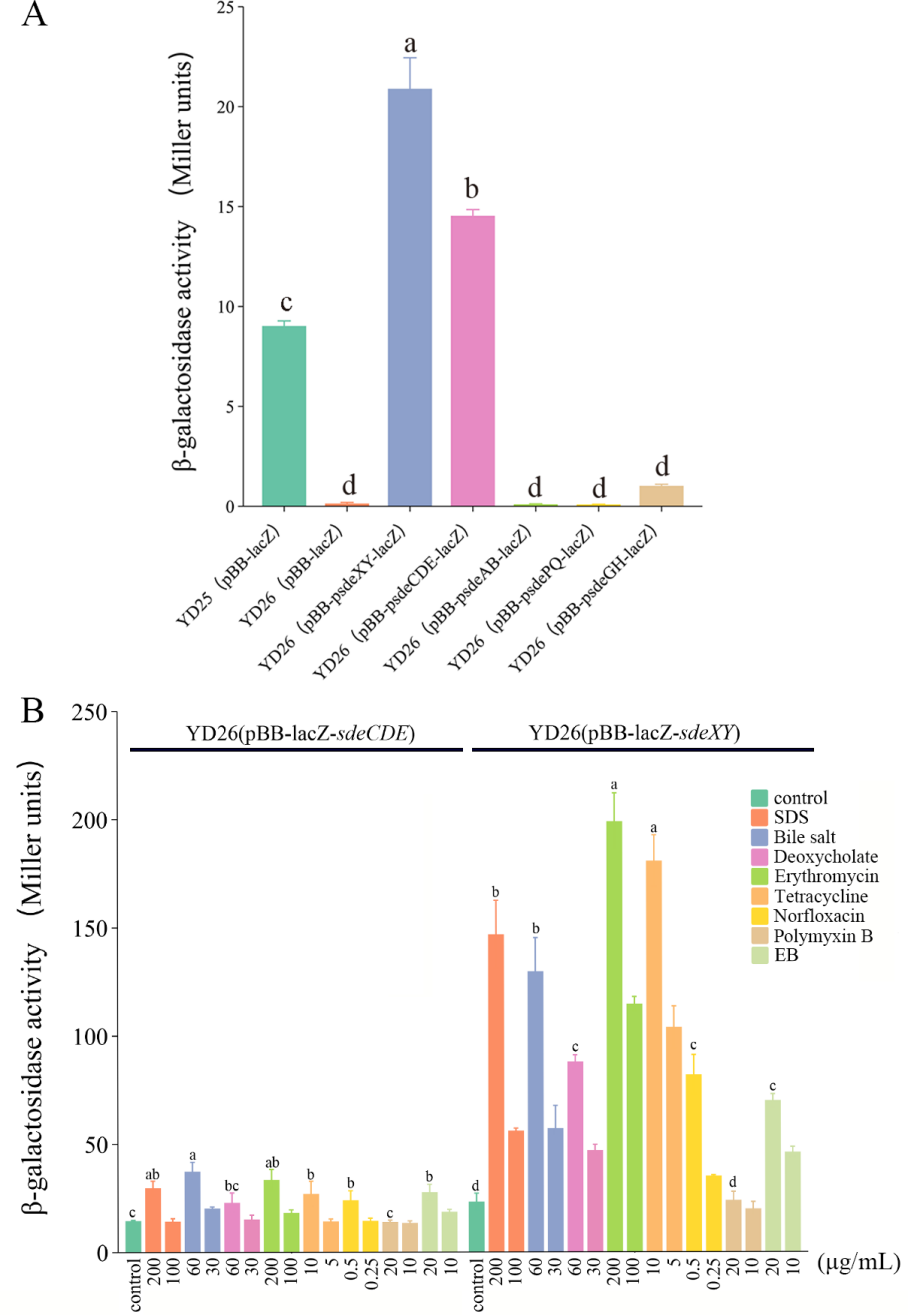
(**A**) Transcriptional activity of RND efflux pump genes in YD25 strains. YD25(pBB-lacZ) demonstrated basal LacZ expression. YD26(pBB-lacZ) served as the negative control because of the complete absence of LacZ activity. Among the five examined RND efflux pumps, the SdeXY and SdeCDE efflux pump genes presented significantly increased transcriptional activity levels. The different superscript letters indicate significant differences (*P* < 0.05) between the experimental groups. (**B**) Efflux activities of SdeXY and SdeCDE toward different substrates. The green column represents the untreated control group, while each remaining color corresponds to a distinct substrate treatment group (two concentration levels per substrate). Statistically significant differences (*P* < 0.05) between the high-concentration treatment groups and the control group are indicated by different superscript letters.

Analysis of the YD25 genome revealed the presence of five RND efflux pumps, namely, SdeXY, SdeAB, SdeCDE, SdeGH, and SdePQ-OmsA (Table S3). To detect the expression levels of these efflux pumps, *lacZ* reporter plasmids containing the promoters of these five genes were constructed. However, due to the presence of an endogenous *lacZ* in the YD25 genome, which results in high background expression. Thus, the *lacZ* was knocked out via homologous recombination in preliminary experiments, generating the YD26 strain for monitoring *lacZ* expression. The enzyme activity of LacZ in YD26 was close to zero and significantly different from that in YD25 (Fig. 2A). The five reporter plasmids were then transformed into YD26, and the data revealed that the level of LacZ expression activated by the *sdeXY* promoter was as high as 20.93 Miller units, followed by that activated by the *sdeCDE* promoter, which was significantly greater than that in the control group. These findings suggested that SdeXY and SdeCDE are constitutively expressed efflux pumps for substrate expulsion in the YD25 strain. The LacZ expression levels activated by the *sdeAB*, *sdeGH*, and *sdePQ-omsA* efflux pump promoters were very low. Therefore, these three types of efflux pumps are likely inducible efflux pumps in YD25 that require specific substrate stimulation.

After stimulation with different substrates, changes in LacZ expression levels activated by the *sdeXY* and *sdeCDE* promoters were reflective of the response of the pump to different substrates (Fig. 2B). With the exception of polymyxin B, the expression of *sdeXY* and *sdeCDE* significantly increased, indicating their involvement with all the other substrates. Furthermore, promoter activity also increased with increasing substrate concentration, which indicated that within a certain range, the expression of *sdeXY* and *sdeCDE* occurred in a concentration-dependent manner. For SdeXY, the most significant upregulation of expression was observed after the addition of the lipophilic antibiotics erythromycin and tetracycline. SdeXY also presented high expression levels under the influence of the anionic surfactants SDS and bile salt. The expression level moderately increased under stimulation with the dye ethidium bromide, the antibiotic norfloxacin, and the anionic surfactant deoxycholate. For the SdeCDE efflux pump, the expression level was most significantly affected by the anionic surfactant bile salt, followed by erythromycin and SDS. Other substrates also caused varying degrees of upregulation of SdeCDE. In summary, the SdeXY and SdeCDE efflux pumps in YD25 can transport a variety of substrates. On the other hand, although they belong to the same RND family, the two efflux pumps still exhibit substrate specificity.

### Effect of local regulatory factors on RND efflux pumps

**Fig 3:**
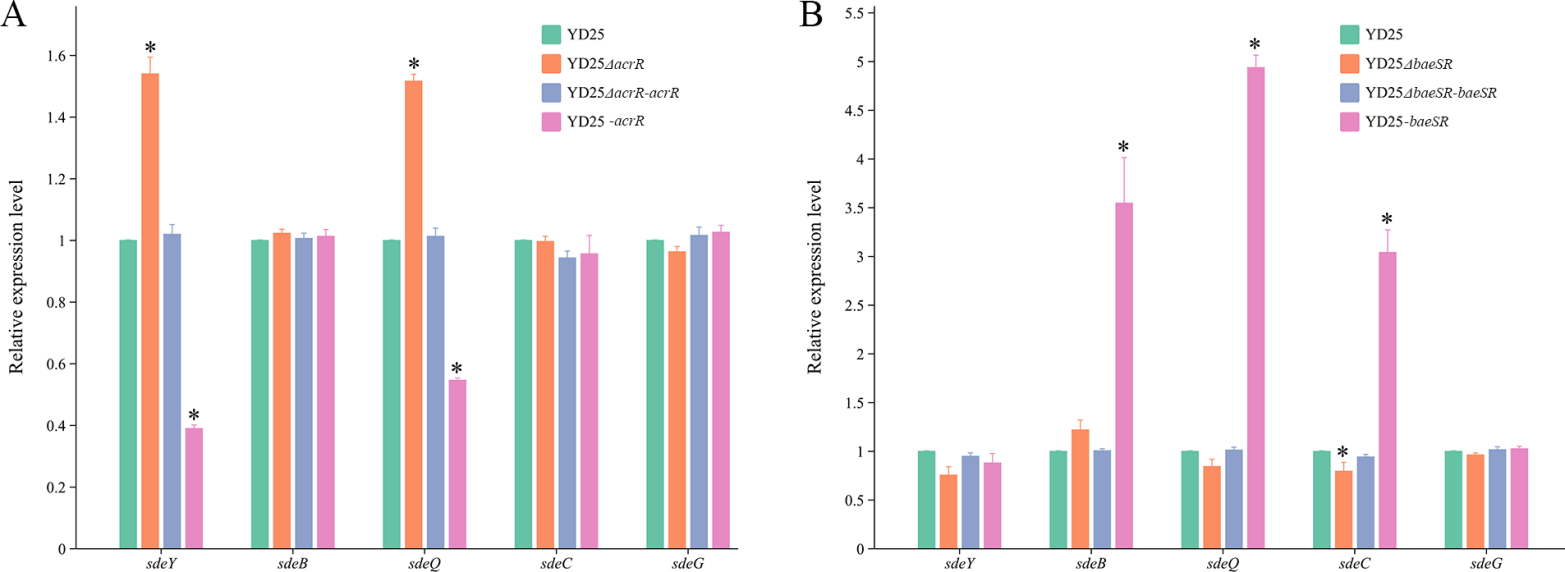
Regulatory effects of the local regulators AcrR (**A**) and BaeSR (**B**) on the expression levels of five RND efflux pump genes. One representative gene was selected from each efflux pump for expression analysis. The green columns represent gene expression levels in wild-type YD25, whereas the orange, blue, and pink columns denote the corresponding knockout, complemented, and overexpression strains, respectively. Asterisks (*) indicate statistically significant differences (*P* < 0.05) between the experimental groups and the YD25 control group.

Bioinformatic analysis revealed that the adjacent regions of the RND efflux pump gene cluster in the YD25 genome have corresponding local regulatory factors (Fig. S3). AcrR is downstream of SdeXY, and its amino acid sequence shares 65% similarity with AcrR from *E. coli*. SdeS and SdeR are situated upstream and downstream of SdeAB, respectively, exhibiting 99% and 96% similarity to SdeS and SdeR from *S. marcescens*. Both BaeS and BaeR are downstream of SdeCDE, and both show 92% similarity to their counterparts in *S. marcescens* (Table S3). However, no corresponding local regulatory factors are found near the gene synthesis clusters of SdeGH and SdePQ-OmsA. Quantitative real-time PCR experiments (Fig. 3) revealed that after *acrR* knockout, the expression of *sdeY* was upregulated, whereas after *acrR* overexpression, the expression of *sdeY* was downregulated. These findings indicate that AcrR is a negative regulatory factor for SdeXY. Interestingly, AcrR plays a negative regulatory role in the expression of the *sdePQ-omsA* efflux pump gene. In the *baeSR* double-knockout strain, *sdeC* was downregulated; after *baeSR* overexpression, the expression level of *sdeC* was significantly increased. This finding indicated that BaeSR is a positive regulatory factor for the SdeCDE efflux pump. Although *baeSR* overexpression also caused the upregulation of *sdeB* and *sdeQ*, the levels of these two genes did not decrease after *baeSR* deletion, suggesting that the effect of BaeSR on *sdeAB* and *sdePQ-omsA* may be indirect.

Compared with the wild-type strain, YD25Δ*acrR* presented increased MICs for five antibiotics, indicating increased antibiotic tolerance in the mutant strain (Table 4). This might be due to the upregulation of *sdeY* in YD25Δ*acrR*, allowing the strain to expel more antibiotics. In YD25-*acrR*, the MICs of the five antibiotics decreased, which is consistent with the downregulation of *sdeY* expression in YD25-*acrR*. However, there was little change in the MIC of ampicillin for both YD25Δ*acrR* and YD25-*acrR*. The bioinformatic screening of the YD25 genome conducted in this study revealed the presence of four β-lactamase genes. Therefore, β-lactamase genes may be involved in influencing YD25 sensitivity to ampicillin rather than just the role of efflux pumps. YD25Δ*baeSR* presented significant decreases in the MICs of ciprofloxacin and novobiocin, indicating increased sensitivity in the mutant strain (Table 4). This could be due to the downregulation of *sdeC* in YD25Δ*baeSR*, leading to a reduced ability of the bacteria to expel antibiotics. In YD25-*baeSR,* the MICs for both ciprofloxacin and novobiocin increased, which was consistent with the elevated expression level of *sdeC* in YD25-*baeSR*. Although no change occurred in the MIC of norfloxacin in YD25Δ*baeSR*, a significant increase in the MIC was observed in YD25-*baeSR*. Considering the changes in the MICs for the other two antibiotics, BaeSR is a positive regulatory factor for the SdeCDE efflux pump overall.

**TABLE 4:** MIC of *acrR* and *baeSR* related strains against different antibiotics^*a*^

Strains	MIC μg/mL (fold)					
	Ampicillin	Tetracycline	Erythromycin	Ciprofloxacin	Norfloxacin	Novobiocin
YD25	512	256	128	8	16	128
YD25*ΔacrR*	550	384 (1.5)	256 (2)	24 (3)	48 (3)	ND
YD25-*acr*R	385	128 (−2)	64 (−2)	4 (−2)	8 (−2)	ND
YD25*ΔbaeSR*	ND	ND	ND	2 (−4)	16 (1)	32 (−4)
YD25-baeSR	ND	ND	ND	64 (−8)	128 (8)	256 (2)

^*a*^ In the fold-change values, a negative number indicates a decreased MIC relative to the control group, suggesting increased bacterial susceptibility to the stimulants, whereas a positive number indicates enhanced bacterial tolerance to the stimulants. "ND" stands for "not detected".

### Effect of global regulatory factors on RND efflux pumps

In addition to local regulatory factors, three classes of global regulatory factors related to efflux pumps have been predicted in the genome of YD25: Rob, SoxS, and RamA. Among them, two Rob factors were identified with similarities reaching 84% and 76% compared with Rob in *E. coli* and *Gibbsiella quercinecans*, respectively; hence, they were named Rob and Roblike (Table S3). The three-dimensional modeling results also revealed that Rob and Roblike share a high degree of similarity in spatial structure (Fig. S4), suggesting potential functional similarities between them. The binding sites for different global regulatory factors in the promoters of RND efflux pump genes were predicted (Table S4). For *sdeXY*, RamA, Rob, and SoxS have corresponding binding sites with likelihood scores that decrease from high to low. For *sdeAB*, binding sites are predicted for both Rob and SoxS. For *sdeCDE*, *sdePQ-omsA,* and *sdeGH*, only a binding site for SoxS is predicted. Owing to the high similarity between Rob and Roblike, they may recognize the same binding sites. Therefore, Roblike might also bind to the promoters of *sdeXY* and *sdeAB*.

After four global regulatory factors were separately knocked out, the sensitivity of the knockout strains to norfloxacin, tetracycline, and erythromycin differed (Table 5). The MICs of YD25Δ*ramA* and YD25Δ*soxS* for the three antibiotics decreased, indicating that SoxS and RamA positively regulate the efflux activity of YD25. YD25Δ*rob* and YD25Δ*roblike* were more sensitive to tetracycline and erythromycin, suggesting that Rob and Roblike also play positive roles, but these two regulatory factors do not affect sensitivity to norfloxacin. The MICs of the three antibiotics in YD25Δ*rob*Δ*roblike* decreased significantly, confirming that Rob and Roblike are positive regulators with overlapping functions. Most complemented strains showed MICs restored to wild-type levels, whereas overexpression strains presented even higher MICs.

**TABLE 5:** MIC of *rob-*, *roblike-, ramA-, and soxS*-related strains against different antibiotics^*a*^

Strains	MIC μg/mL (fold)		
	Tetracycline	Erythromycin	Norfloxacin
YD25	256	128	16
YD25Δ*rob*	128 (−2)	64 (−2)	16 (1)
YD25Δ*roblike*	128 (−2)	32 (−4)	16 (1)
YD25Δ*ramA*	128 (−2)	32 (−4)	8 (−2)
YD25Δ*soxS*	64 (−4)	64 (−2)	8 (−2)
YD25Δ*rob*Δ*roblike*	32 (−8)	8 (−16)	8 (−2)
C-YD25Δ*rob*(*rob*)	256 (1)	256 (2)	32 (2)
C-YD25Δ*roblike*(*roblike*)	128 (−2)	128 (1)	32 (2)
C-YD25Δ*ramA*(*ramA*)	256 (1)	128 (1)	16 (1)
C-YD25Δ*soxS*(*soxS*)	256 (1)	128 (1)	16 (1)
YD25-*rob*	>1,000 (>3)	384 (3)	64 (4)
YD25-*roblike*	>1,000 (>3)	>512 (>4)	64 (4)
YD25-*ramA*	512 (2)	384 (3)	48 (3)
YD25-*soxS*	>1,000 (>3)	256 (2)	32 (2)

^*a*^ In the fold-change values, a negative number indicates a decreased MIC relative to the control group, suggesting increased bacterial susceptibility to the stimulants, whereas a positive number indicates enhanced bacterial tolerance to the stimulants.

**Fig 4:**
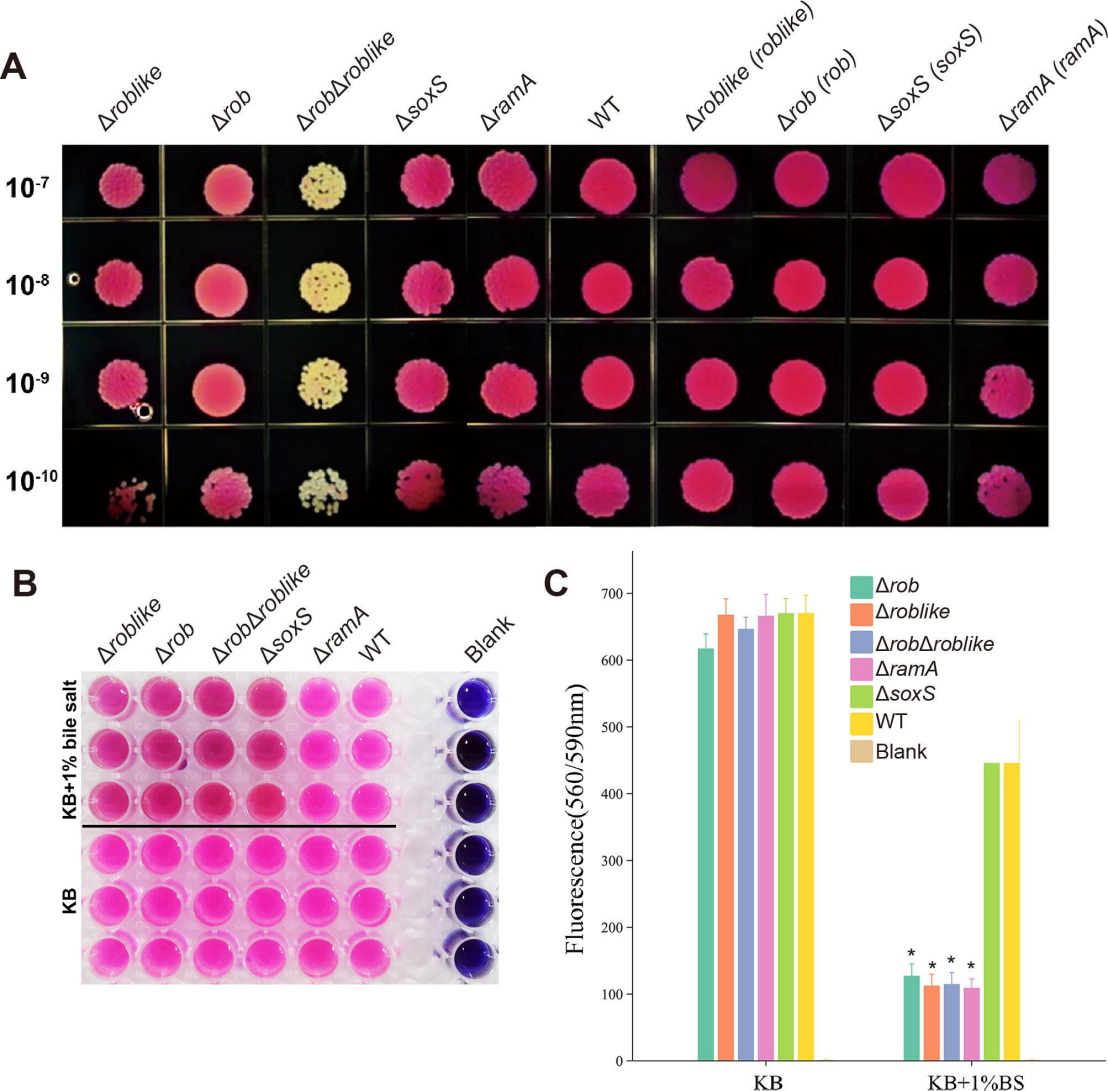
(**A**) SDS tolerance of regulator-related strains. The serial dilutions are indicated in the left panel. The identities of the strains are indicated above. Compared with the wild-type strain, YD25Δ*rob*Δ*roblike* exhibited progressively reduced bacterial growth starting from the 10^−7^ dilution. YD25Δ*roblike* showed markedly decreased growth at the 10^−10^ dilution. YD25Δ*rob*, YD25Δ*soxS*, and YD25Δ*ramA* also exhibited reduced growth at the 10^−10^ dilution, although to a lesser extent than YD25Δ*roblike*. (**B and C**) Bile salt tolerance of regulatory factor-associated strains. Panel **B** shows the resazurin staining results of relevant strains cultured in both KB medium and KB medium containing 1% bile salt. "Blanks" represent the negative control. Panel **C** was generated on the basis of the fluorescence values of different strain. Asterisks (*) indicate statistically significant differences (*P* < 0.05) between the knockout strains and the YD25 control.

In addition to MIC testing, tolerance to SDS and bile salts, two types of surfactants, was also compared between the knockout and wild-type strains. YD25 exhibited strong tolerance, with bacterial cultures diluted from 10^−7^ to 10^−10^ able to grow normally under 2% SDS. Knockout strains showed decreased tolerance to SDS to a certain extent and became even less tolerant as the bacterial culture concentration decreased. Specifically, YD25Δ*rob*Δ*roblike* presented the lowest tolerance to SDS at equivalent concentrations. The complemented strains showed tolerance to 2% SDS, which was consistent with that of the wild type. Resazurin staining was used to assess the effect of bile salts on bacterial activity. With the exception of YD25Δ*soxS*, the growth of the other knockout strains was inhibited by 1% bile salts (Fig. 4). These results further confirmed that all four global factors positively regulate efflux activity in YD25.

**Fig 5:**
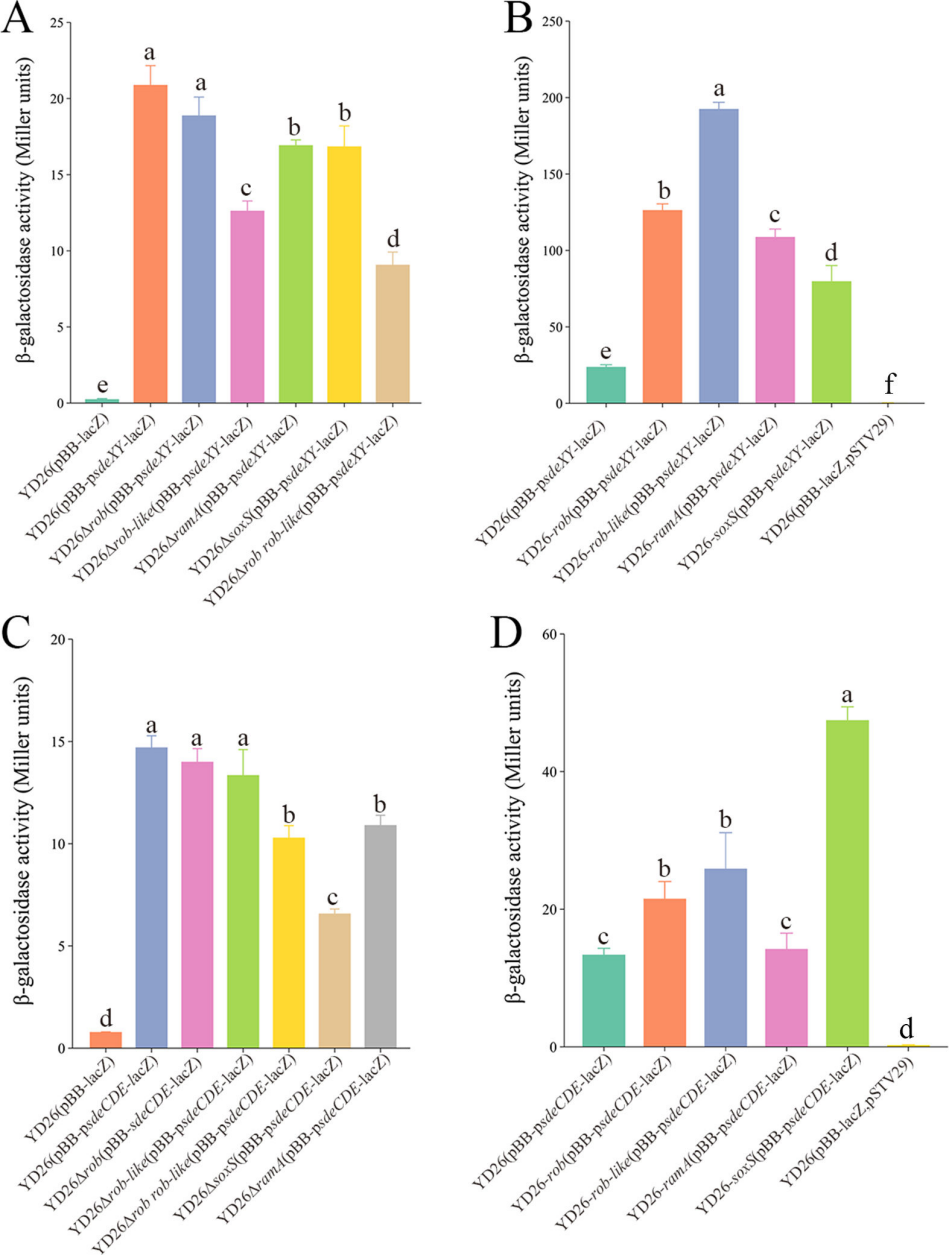
Transcriptional levels of the *sdeXY* (**A**) and *sdeCDE* (**C**) efflux pump genes in regulatory factor knockout strains. YD26(pBB-lacZ) served as the negative control because of the complete absence of LacZ activity. YD26(pBB-p*sdeXY*-lacZ) represents the *sdeXY* transcription level in the wild-type strain, and YD26(pBB-p*sdeCDE*-lacZ) represents the *sdeCDE* transcription level in the wild-type strain. Statistically significant differences (*P* < 0.05) between each strain are indicated by different superscript letters. The transcriptional levels of the *sdeXY* (**B**) and *sdeCDE* (**D**) efflux pump genes in regulatory factor-overexpressing strains. YD26(pBB-p*sdeXY*-lacZ) and YD26(pBB-p*sdeCDE*-lacZ) served as the positive controls. YD26(pBB-lacZ,pSTV29) served as the negative control. Statistically significant differences (*P* < 0.05) between each strain are indicated by different superscript letters.

The effects of global regulatory factors on the expression of efflux pump genes were analyzed through Miller assays. For SdeXY, knocking out *rob* slightly affected the expression level of the efflux pump promoter (Fig. 5A). Knocking out *roblike*, *ramA*, or *soxS* reduced *sdeXY* expression, with *roblike* having the strongest effect. Although knocking out *rob* alone did not significantly affect *sdeXY* expression, the YD26Δ*rob*Δ*roblike* double mutant showed a synergistic effect, leading to significant downregulation of *sdeXY*. Moreover, in the overexpressed strains, the expression level of *sdeXY* was significantly upregulated, with the regulatory factors having the following effects from high to low: Roblike, Rob, RamA, and SoxS (Fig. 5B). In summary, all four regulatory factors positively regulate the expression of the *sdeXY* efflux pump gene, with Roblike having the strongest regulatory effect and Rob/Roblike being functionally redundant.

Compared with the control strain, knocking out *rob* and *roblike* had minimal impact on *sdeCDE* expression, indicating that Rob and Roblike are not its major regulators (Fig. 5C). In YD26Δ*rob*Δ*roblike*, decreased *sdeCDE* expression indicated positive regulatory roles for Rob and Roblike, despite their single knockouts having no effect. Knocking out *ramA* and *soxS* resulted in a significant decrease in *sdeCDE* expression, with SoxS having the strongest positive regulatory effect on *sdeCDE* among the tested regulatory factors. Moreover, in the overexpressed strains, the expression level of *sdeCDE* was upregulated, with the regulatory factors having the following effects from high to low: SoxS, Roblike, and Rob (Fig. 5D). In summary, SoxS positively regulates the expression of *sdeCDE*, whereas RamA, Rob, and Roblike have slight but nonsignificant effects on the expression of *sdeCDE*.

### Effects of oxyresveratrol on the SdeXY efflux pump

Following the knockout of the efflux pump SdeXY, the MICs of the host strain against the four antibiotics decreased by 2-fold to 4-fold, whereas no change was observed in the MIC for erythromycin (Table 6). Although the reduction in MICs was not substantial, the overall trend indicated decreased antibiotic resistance in the strain, suggesting impaired antibiotic efflux capability due to the absence of SdeXY. Combination experiments were conducted using five antibiotics with oxyresveratrol. The MICs of these antibiotics for wild-type YD25 decreased, which is consistent with previous results. Notably, the MICs of the YD25Δ*sdeXY* strain remained unchanged before and after the addition of oxyresveratrol. This indirectly confirms that oxyresveratrol acts as an inhibitor of the SdeXY efflux pump. In this case, since SdeXY was absent, oxyresveratrol lacked its target and thus had no effect, leaving the antibiotic resistance of the strain unaltered.

**TABLE 6:** MIC of YD25Δ*sdeXY* against different antibiotics^*a*^

Strains		MIC μg/mL (fold)				
		Ampicillin	Kanamycin	Tetracycline	Erythromycin	Chloramphenicol
YD25	Without oxyresveratrol	512	32	256	128	128
YD25Δ*sdeXY*		256 (−2)	16 (−2)	128 (−2)	128 (1)	32 (−4)
YD25	With oxyresveratrol	256 (−2)	2 (−16)	64 (−4)	64 (−2)	8 (−16)
YD25Δ*sdeXY*		256 (−2)	16 (−2)	128 (−2)	64 (−2)	32 (−4)

^*a*^ In the fold-change values, a negative number indicates a decreased MIC relative to the control group, suggesting increased bacterial susceptibility to the stimulants, whereas a positive number indicates enhanced bacterial tolerance to the stimulants.

## DISCUSSION

Bacteria develop resistance via three primary mechanisms: intrinsic, acquired, and adaptive resistance. Intrinsic resistance is an inherent property of host bacteria, involving antibiotic degradation or modification by resistance genes, reduced membrane permeability, or active expulsion of toxic substrates. Acquired resistance arises from horizontal gene transfer (e.g., plasmids) or gene mutations. Adaptive resistance, also known as phenotypic resistance, is a temporary, non-heritable response to environmental stimuli, which reverses once the trigger is removed. Analysis of the genomes of 32 strains belonging to the *Serratia* genus revealed that both environmental and clinical isolates possess intrinsic antibiotic tolerance genes (ARGs) in their genomes (5). Genomic analysis confirms that YD25’s strong antibiotic tolerance is intrinsic, primarily due to its lack of plasmids and a genome enriched with 41 efflux pump genes.

The efflux pump is one of the most important contributors to the intrinsic drug resistance phenotype (22) because of its ability to recognize various toxic substances, such as detergents, fatty acids, heavy metals, bile salts, dyes, and antibiotics. SdeXY is the major contributor to intrinsic multidrug resistance in *S. marcescens*, resulting in resistance to various antibiotics and xenobiotics in both clinical and environmental isolates (8, 11, 23). Another significant player in multidrug resistance in *S. marcescens* is the SdeAB efflux pump; however, its substrate range is smaller than that of SdeXY, and its substrate selectivity and contribution to drug resistance are strain-specific (11, 16). SdeGH and SdePQ-OmsA in *S. marcescens* Db10 have broad substrate specificity and can expel norfloxacin, novobiocin, benzalkonium chloride, deoxycholate salts, EB, and SDS (23). The associations of other *Serratia* RND efflux pumps with the multidrug resistance phenotype are relatively weak. In *S. marcescens* UOC-67, SdeCDE is associated only with resistance to neomycin (16), whereas in *S. marcescens* Db10, SdeCDE is correlated with resistance to norfloxacin and SDS (11). Similarly, SdeIJ and SdeNO from Db10 are unrelated to antibiotic resistance, with SdeIJ showing some resistance to benzalkonium chloride and SdeNO having some resistance to SDS (11). In strain YD25, SdeXY shows resistance to multiple antibiotics and xenobiotics, which is consistent with previous reports. Notably, SdeCDE also significantly contributes to multidrug resistance in YD25, resulting in resistance to three antibiotics—norfloxacin, erythromycin, and tetracycline—as well as resistance to SDS, sodium deoxycholate, taurocholic acid, and EB (Fig. 2B). Combining the results of this study with the high expression levels of both SdeXY and SdeCDE in YD25 compared with that of SdeAB (Fig. 2A), SdeXY is speculated to be constitutively expressed in the strains of *Serratia*. This is consistent with previous research reporting that SdeXY is a major contributor to inherent multidrug resistance in *S. marcescens* strains. Moreover, the expression of SdeAB and SdeCDE varies among different strains of *Serratia*; in YD25, high expression of SdeCDE complements low expression of SdeAB, contributing to multidrug resistance in host bacteria. Similar to *S. marcescens* UOC-67, strain-specific differences in expression levels, substrate selectivity, and contributions to drug resistance are also found for SdeAB (16).

**Fig 6:**
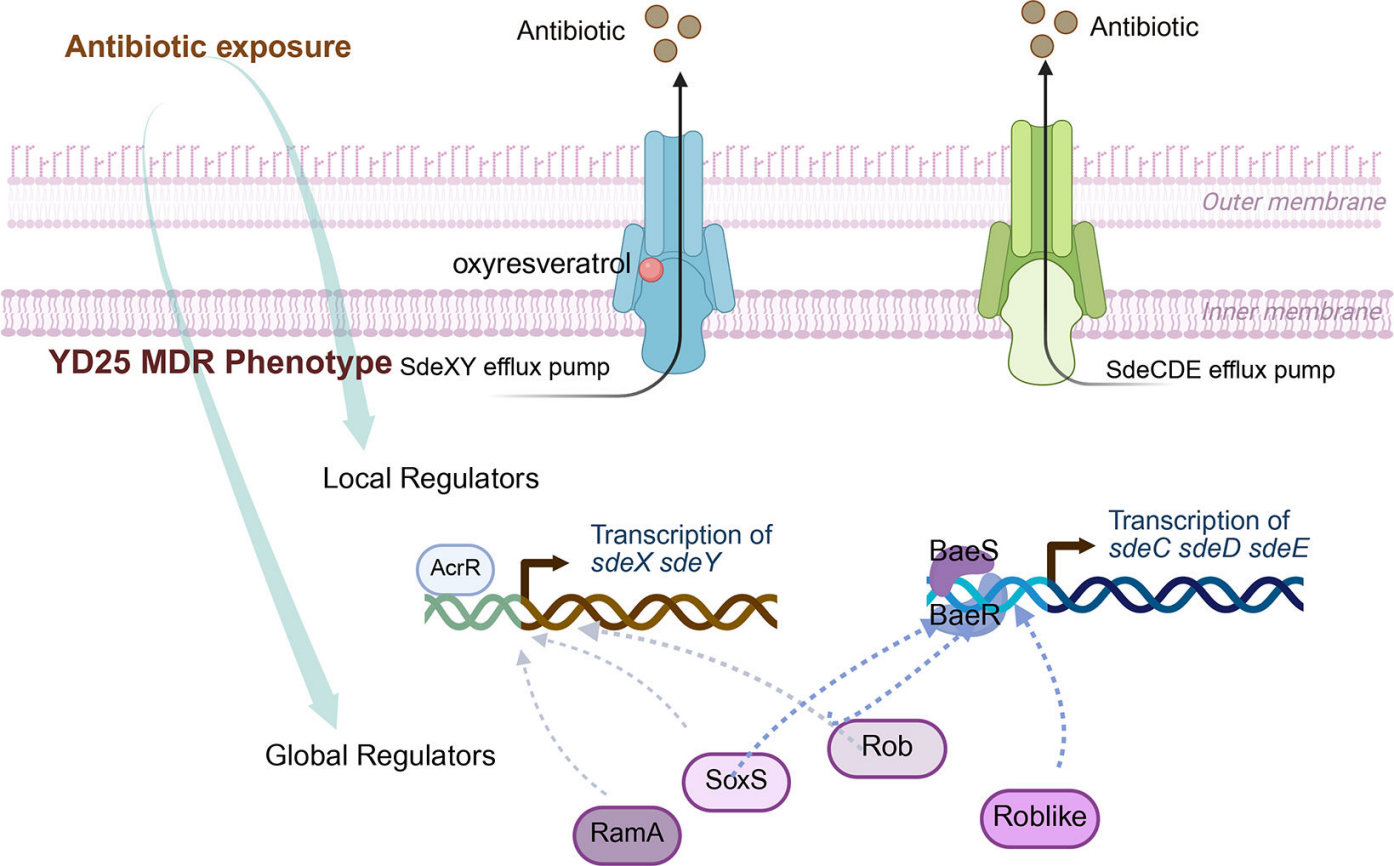
Graphical summary of the research findings. YD25 contains two constitutively expressed RND efflux pumps, SdeXY and SdeCDE. The expression of efflux pump genes is regulated through a hierarchical network, spanning from global to local regulators. Oxyresveratrol functions as an efflux pump inhibitor specifically targeting SdeXY. By targeting the SdeY, oxyresveratrol restores the susceptibility of YD25 to multiple antibiotics.

Experimental results confirmed that the gene encoding the negative regulator AcrR was located adjacent to the *sdeXY* efflux pump genes in YD25, demonstrating high consistency with the AcrAB-AcrR system in *E. coli* in terms of both genomic structure and function (24). SdePQ-OmsA in YD25 shows high similarity to the CmeABC efflux pump in *Campylobacter jejuni*. In the *C. jejuni* genome, the side region of the CmeABC synthesis cluster contains a regulatory factor called CmeR, which can downregulate the expression of *cmeABC* (25). No gene encoding a CmeR-like factor was found near SdePQ-OmsA in YD25. However, interestingly, AcrR not only negatively regulates SdeXY but also has a negative regulatory effect on SdePQ-OmsA in YD25. AcrR and CmeR both belong to the TetR family of regulatory factors, and they prevent downstream gene transcription by binding to specific sequences in the target gene promoter region (26). While the AcrR recognition sequence is absent in the *sdePQ-omsA* promoter region, suggesting that the effect of AcrR on SdePQ-OmsA is indirect (Fig. 6). In YD25, SdeCDE shares some similarity with MdtABC in *E. coli*. In *E. coli*, BaeSR functions as a two-component regulatory system and upregulates *mdtABC* (27). In *Acinetobacter baumannii*, BaeSR upregulates the expression of AdeAB, a type of RND pump, to confer resistance to antibiotics such as tigecycline (28). In *Salmonella enterica serovar Typhimurium*, BaeSR also modulates the expression of AcrD and MdtABC (29). Similarly, near *sdeCDE* in YD25, BasSR acts as a positive regulator of *sdeCDE* (Fig. 6). Collectively, the action of local regulators on efflux pumps in YD25 aligns with the established model for *Serratia* bacteria.

The global regulator RamA enhances efflux pump activity in YD25, demonstrating a conserved function from its positive regulation of the AcrAB-TolC system in *S. marcescens* RM66262 (17). In *E. coli* and *Salmonella enterica*, overexpression of the global regulator SoxS upregulated *acrAB*, enhancing efflux pump activity and conferring drug resistance to the host bacteria (30, 31). Similarly, SoxS also upregulates efflux pumps in YD25, which represents the first report of this regulatory function of SoxS in a *Serratia* strain.

Unlike its single-copy role in mediating broad-spectrum resistance via the AcrAB efflux pump in bacteria like *E. coli*, *Enterobacter cloacae*, and *Salmonella typhimurium* (32–35), the global regulator Rob has two functionally redundant homologs (Rob and Roblike) in strain YD25 that jointly confer multidrug resistance (Fig. 6). This represents the first report that two functional Rob homologs have been identified in a single bacterial strain. Despite 50% sequence similarity and analogous 3D structures between them, Rob and Roblike in YD25 show distinct external homology: Rob to *E. coli* MG1655 (84%) and *Yersinia pestis* KIM10+ (87%), and Roblike to *Gibbsiella quercinecans* FRB97 (89%) and *Chania multitudinisentens* RB-25 (86%). Bioinformatic analysis of the available 175 *Serratia* genomes in GenBank (taxid: 2741499) revealed that 161 strains possess both *rob* and *roblike* genes, one strain has only *rob*, and 13 strains lack both. Furthermore, the genomic contexts of *rob* and *roblike* are conserved across the 161 positive strains (Fig. S4). The divergent origins yet widespread co-occurrence of both Rob and Roblike across the *Serratia* genus led us to hypothesize that these two genes were acquired from distinct sources early in its evolutionary history. This, in turn, further suggests that the mechanism—whereby these two homologs function cooperatively to enhance efflux pump expression and confer stronger drug resistance to the host—is likely universal within *Serratia*.

Based on experimental confirmation that CPZ and RSP act as inhibitors of RND and MFS efflux pumps, respectively, CPZ was selected as the positive control in this study (36). However, it has also been reported that RSP can inhibit the AcrB transporter (37). Therefore, in the clinical treatment of *Serratia* infection, the use of EPIs (e.g., RSP or CCCP) in combination with antibiotics is a potential therapeutic strategy to counteract resistance and prevent its spread. Research demonstrated that RSP reduced resistance to disinfectants DDAC and BAC in both multidrug-resistant and standard *Serratia* strains. In contrast, CCCP only reversed DDAC resistance in the standard strain and was ineffective against the resistant isolate (38). This result indicates that different EPIs may not have the same effect on different *Serratia* strains. Researchers have been working on discovering new, highly effective, and low-toxicity EPIs. For example, trimethoprim and epinephrine can enhance the sensitivity of a range of *Enterobacteriaceae*, including *S. marcescens* NCTC 2847 and *P. aeruginosa* NCTC 10662, to antibiotics to some extent (39). However, research on EPIs against *Serratia* is relatively limited at present. On the basis of the theory of “food‒medicine homology,” plant-derived antimicrobials are among the important sources for screening EPIs (40, 41). For example, *Cinnamomum verum* oil can inhibit the RND efflux pump in *A. baumannii* (42); pyrrole-based compounds inhibit RND-type efflux pump activity in *E. coli* and *P. aeruginosa* through binding to their respective pump components, AcrB and MexB (43); and p-coumaric acid and its derivatives were selected from 328 plant metabolites to inhibit the RND efflux pump of *P. aeruginosa* (44). Molecular docking reveals that all four pyrrole derivatives bind to the same phenylalanine-rich substrate-binding pocket in both AcrB and MexB proteins, stabilized by common interactions with Phe128, Phe178, Phe610, and Phe628. Furthermore, comparative analysis of the binding of AcrB and MexB with the known inhibitor PAβN indicates that it also occupies the same site and shares common interacting residues (43). Oxyresveratrol binds to SdeY—a homolog of AcrB and MexB—yet at a completely distinct binding site with different interacting residues. BLI studies confirmed the binding of pyrrole derivatives to both AcrB and MexB, with molecular docking predicting their binding energies to range from −10.168 to −8.427 kcal/mol. In comparison, the binding energy of oxyresveratrol to SdeY was −7.39 kcal/mol, indicating a strong and stable interaction—despite the absence of commonality in their binding sites.

The discovery of a growing number of plant-derived natural compounds, such as oxyresveratrol, as potent multidrug resistance inhibitors, opens a new frontier in antimicrobial research. These compounds play a critical role in developing next-generation therapeutics, especially in synergistic combination with conventional antibiotics, by effectively neutralizing bacterial efflux mechanisms. With the rapid refinement of computational tools like AlphaFold 3, which enables highly accurate protein-ligand modeling, molecular docking technology can now efficiently screen and identify novel EPIs from extensive libraries of plant-derived compounds. This approach represents a profoundly promising strategy to address the escalating global challenge of drug-resistant bacteria.

### Conclusion

This study is the first to perform bioinformatics analysis of the YD25 genome to identify multiple antibiotic resistance-related genes, including efflux pump genes of five families. Screening of natural compounds revealed that oxyresveratrol specifically inhibits the RND efflux pump SdeXY. Further research revealed the presence of five RND efflux pumps in YD25, with SdeXY and SdeCDE being constitutively expressed. These efflux pump gene clusters are regulated by the local regulators AcrR and BaeSR. Genomic analysis also identified global regulators, including RamA, SoxS, and Rob. We are the first to report the positive regulatory role of SoxS on efflux pumps in *Serratia*. Another novel finding is that YD25 contains a homolog of Rob, named Roblike. Although *roblike* and *rob* have different origins, they share the same function—both positively regulate efflux pumps (Fig. 6). YD25 represents a new species within the *Serratia* genus and shares a close evolutionary relationship with *S. marcescens*. Therefore, studying the resistance mechanisms of YD25 can not only elucidate the resistance mechanisms of this new species but also contribute to understanding the multidrug resistance of *S. marcescens*, providing valuable information for rational clinical drug use.

## MATERIALS AND METHODS

### Bioinformatic analysis and molecular docking

The resistance-related genes in the YD25 genome were predicted using the Comprehensive Antibiotic Resistance Database (CARD) RGI (https://card.mcmaster.ca/analyze/rgi RGI 6.0.3, CARD 3.2.8). Homology modeling of efflux pump proteins was performed based on the Swiss-model database (https://www.swissmodel.expasy.org/interactive). Molecular docking between the proteins and tens of thousands of natural compounds in the Traditional Chinese Medicine Database@Taiwan database (https://zinc.docking.org/catalogs/tcmnp/) was carried out using Autodock/Vina and PyRx software.

Amino acid sequences of RND-type efflux pumps that have been reported in the NCBI database were obtained and used as references to annotate the YD25 whole genome sequence in SnapGene. The local regulatory factors of the RND efflux pump were screened based on genome annotation in the upstream and downstream regions of the efflux pump gene. Amino acid sequences of relevant proteins were obtained from the NCBI database, and protein homology analysis was performed using ClustalW (https://www.genome.jp/tools-bin/clustalw). Furthermore, global regulatory factors Rob, SoxS, and RamA were also predicted by homology analysis. The promoter region, approximately 1,000 bp upstream of the RND efflux pump gene, was obtained, and the binding sites of Rob, SoxS, and RamA were analyzed using PRODORIC2 (http://www.prodoric2.de).

### Determination of the minimum inhibitory concentration of antibiotics

Antibiotics were dissolved in MH medium to different final concentrations. YD25 was inoculated into MH medium and cultured until the OD_600_ reached between 0.4 and 0.6. The bacterial culture was then diluted with MH medium to 10^6^ CFU/mL. Subsequently, 200 μL of the bacterial culture was mixed with 200 μL of antibiotic solutions of different final concentrations and incubated at 37°C for 20 h. The absorbance values at OD_600_ of each sample were measured to calculate the MIC of each antibiotic. The experiment was repeated at least three times.

### Fluorescent dye accumulation and efflux experiment

Hoechst 33342 is a bisbenzimide dye that is water-soluble and has low cytotoxicity. Outside of cells, this dye exhibits weak fluorescence; however, upon entering cells, its fluorescence signal becomes very strong. This property makes Hoechst 33342 suitable for real-time monitoring of substrate accumulation and efflux in bacterial cell suspensions. YD25 was inoculated into LB medium and cultured until reaching an OD_600_ of 0.6. The cells were then collected by centrifugation, washed twice with PBS buffer, and finally resuspended in PBS solution containing 0.4% glucose to achieve an OD_600_ of 0.4. Hoechst 33342 was added to a final concentration of 2.5 μmol/L, and different natural compounds were separately added to reach a final concentration of different sub-MIC. On the other hand, the YD25 cell suspension was prepared as previously described, and the Hoechst 33342 was added to a final concentration of 2.5 μmol/L. The mixture was incubated at 25°C for 60 min to allow sufficient accumulation of the fluorescent dye within the cells. Subsequently, collect the cells by centrifugation, wash them with PBS solution containing 0.4% glucose, and resuspend the cells. Then, different natural compounds were added separately to achieve a final concentration of different sub-MIC. The mixture was subjected to real-time fluorescence accumulation monitoring at 37°C, with excitation at 355 nm and emission at 465 nm. Data were recorded every 60 s for a total of 30 min. CPZ at 1/2× MIC was used as the positive control, the YD25 culture as the negative control, and PBS solution as the blank control. The experiment was repeated three times for validation.

### Combined antibacterial experiment of natural efflux pump inhibitors and antibiotics

Oxyresveratrol was dissolved in MH medium to a final concentration of 1/10× MIC; evernic acid and morin were dissolved separately to a final concentration of 1×MIC. CPZ was also dissolved in MH to 1× MIC and used as a positive control. YD25 was inoculated into MH medium and cultured until the OD_600_ was between 0.4 and 0.6. The cells were collected by centrifugation and then resuspended in MH medium containing different EPIs to a concentration of 10^6^ cfu/mL. MH medium without EPI was used as a control group. The serial 2-fold dilution of various antibiotics was prepared with MH medium. Then, 200 μL of bacterial culture and 200 μL of antibiotic-MH medium of different final concentrations were mixed and cultured at 37°C for 20 h. The absorbance value of each sample was detected at OD_600_, and the MIC value of each antibiotic in the presence of sub-MIC EPI was calculated. Also, the fold change of MIC compared with the MIC value without inhibitor was calculated too. The experiment was repeated no less than three times.

### Checkerboard synergy assay

Oxyresveratrol at 1× MIC and the individual antibiotics at 1× MIC were used as stock solutions, from which serial dilutions were prepared in MH medium. The 96-well plate was prepared with serial antibiotic dilutions in columns and varying compound concentrations in rows. Each well was finally inoculated with 100 μL of 5 × 10^6^ cfu/mL bacterial culture and incubated for 20 h at 37°C. The FICI was calculated as follows:

FICI = FIC_A_+ FIC_B_

where FICI_A_ = MIC of compound A in combination/MIC of compound A alone, and FICI_B_ = MIC of compound B in combination/MIC of compound B alone

The FICIs were obtained from three independent experiments and interpreted as previously described: synergy (FICI ≤ 0.5), additive (0.5 < FICI ≤ 1), no interaction (1 < FICI ≤ 4), and antagonism (FICI > 4) (45).

### Construction of reporter plasmids and promoter activity assay

The backbone of pBBR1MCS-5 plasmid was amplified, and at the same time, the lacZ gene fragment was amplified using YD25 genome as a template. These two fragments were connected by the In-Fusion method to obtain the pBB-lacZ vector. The promoter region of efflux pump genes *sdeXY*, *sdeAB*, *sdeCDE*, *sdeGH,* and *sdePQ* was amplified using YD25 genome as a template. Then, each of these promoter fragments was connected with the above two fragments by In-Fusion method to obtain the lacZ reporter plasmids with different promoters, pBB-*psdeXY*-lacZ, pBB-*psdeAB*-lacZ, pBB-*psdeCDE*-lacZ, pBB-*psdeGH*-lacZ, and pBB-*sdePQ*-lacZ (Table S5). Positive clones were verified by PCR screening and sequencing. All PCRs were performed using 2 × Phanta Max Master Mix (Vazyme, China), and In-Fusion was performed using the NovoRec plus One step PCR Cloning Kit (Novaprotein, China).

The *lacZ* gene in YD25 was knocked out to construct the strain YD26, which had the same physiological and metabolic characteristics of YD25 (unpublished data). The lacZ reporter plasmids were transformed into the YD26 strain, respectively. Meanwhile, the pBB-lacZ vector was transformed into YD25 and YD26, respectively, as the control system. The modified Miller assay was carried out to determine β-galactosidase activity. In brief, a single colony from each strain, streaked on an LB plate, was inoculated into LB broth and cultured for 8–10 h. The cells were then collected by centrifugation at 4°C and resuspended in pre-chilled Z buffer to an OD_600_ of 0.8–1.0. Then, 200 μL of the cell suspension was mixed with 100 μL chloroform and 50 μL 0.1% SDS and incubated at 37°C for 15–20 min. Subsequently, 200 μL of 4 mg/mL ONPG was added, and the mixture was incubated at 37°C, with the time of substrate addition accurately recorded. When the solution turned yellow, 500 μL of 1 mol/L Na_2_CO_3_ was added to stop the reaction, and the time of cessation was accurately recorded. After centrifugation, 200 μL of the supernatant was added to a 96-well plate, and the absorbance at 420 nm was measured. β-galactosidase activity was calculated according to the following formula. The experiment was repeated three times.

Calculate Miller Units as:


$$1000*\frac{\left(Abs_{420}\right)}{\left((Abs_{600}\text{ of culture sampled})*(\text{volume [0.02 mL]})*(\text{reaction time})\right)}$$

The reaction time (min) is the cessation time minus the start time; the volume (mL) is the volume of cell suspension taken; Abs_420_ is the absorbance value of the reaction product; and Abs_600_ is the absorbance value of the bacterial suspension.

### Identification of efflux pump substrates

Norfloxacin, tetracycline, erythromycin, sodium deoxycholate, bile salts, sodium dodecyl sulfate, ethidium bromide, and polymyxin B were diluted in LB medium, respectively, to different final concentrations. Strains containing the reporter plasmids were streaked on LB plates, and a single colony was picked and cultured in LB broth for 4 h. This pre-culture was then subcultured at a 1% inoculum into the prepared LB medium containing various substrates and cultured at 37°C for 20 h. At the same time, strains inoculated into LB medium were used as a control. Subsequently, β-galactosidase activity of each test strain was determined using the same method as described above. The experiment was repeated three times.

### Construction of knockout and overexpression strains of local regulators

Using the YD25 genome as a template, specific fragments of each regulatory factor were amplified (Table S6). The PCR products were purified using the HiPure Gel Pure DNA Mini Kit (MGio, China) after double digestion with *Kpn* I and *Sal* I. The knockout plasmid pKNOCK-Gm was also double digested and purified, and then connected with each insert and transformed using the DNA Ligation Kit (TaKaRa, China). The positive knockout plasmids were screened by sequencing. The knockout plasmid was transferred into the recipient strain YD25 by triparental mating, with *E. coli* DH5α containing pRK2013 as the helper strain. The positive clones were screened on LB solid plates containing 50 μg/mL kanamycin and 25 μg/mL tetracycline, and the knockout strain was confirmed by sequencing.

Using the YD25 genome as a template, specific coding region fragments of each regulatory factor were amplified, and the PCR products were purified after double digestion with *Hin*d III and *Kpn* I. The plasmid pSTV29-Cm was also double digested and purified, then connected with the fragments and transformed, and positive plasmids were screened by sequencing. The expression plasmid was transferred into wild-type YD25 or the corresponding gene knockout strain by electroporation. The positive clones were screened on LB solid plates containing 300 μg/mL chloramphenicol, and the overexpression or the complemented strain was confirmed by sequencing.

### Construction of knockout and overexpression strains of global regulators

Using the suicide vector pKnock-Gm-I-SceI(Rha)-CcdB (constructed and stored in the laboratory), the global regulatory factor genes of YD25 were knocked out through two rounds of allele exchange-homologous recombination relying on I-SceI cutting of the genome and the reverse screening effect of CcdB toxin protein. First, the pKnock-Gm-I-SceI(Rha)-CcdB plasmid was used as a template for PCR amplification to linearize it, with primers pknock-Gm-F/pknock-Gm-R (Table S7). Next, a pair of primers was designed upstream and downstream of the coding region of the global regulatory factor genes, and the upstream and downstream homologous arms were amplified by PCR. The plasmid and homologous arm fragments were purified, and the three fragments were connected using the In-Fusion method. After transformation, the positive knockout plasmids were screened by sequencing. The knockout plasmid was then transferred into *E. coli* S17-1-λpir competent cells and transferred into the recipient strain YD25 by biparental mating. The knockout strain was screened by PCR and verified by sequencing.

Using the YD25 genome as a template, specific coding region fragments of each regulatory factor were amplified. The pSTV29-Cm plasmid was also linearized by PCR, with primers pBBR1MCS-5-F/pSTV29-R. The two fragments were connected using the In-Fusion method, and the positive expression plasmids were screened by sequencing. The expression plasmid was transferred into the recipient strain YD25 and the corresponding knockout strain by electroporation, and positive clones were screened on LB solid plates containing 300 μg/mL chloramphenicol. The overexpression strain and complementation strain were confirmed by sequencing.

### Determination of strains’ resistance impacted by regulatory factors

The knockout, overexpression, and complementation strains, as well as YD25, were inoculated into MH medium and cultured until reaching an OD_600_ between 0.4 and 0.6. The bacterial suspension was then diluted to 10^6^ cfu/mL in MH medium. Various antibiotics were dissolved in MH medium and serially diluted 2-fold to prepare test solutions of different final concentrations; 200 μL of bacterial suspension was mixed with 200 μL of solutions containing different final concentrations of antibiotics and incubated at 37°C for 20 h. The positive control group consisted of bacterial suspension mixed with MH medium without antibiotics, while the negative control group consisted of MH medium alone. The absorbance values of each sample were measured at OD_600_ to calculate the MIC values for each antibiotic. The experiment was repeated thrice.

### Determination of efflux pump gene expression level influenced by regulatory factors

The knockout, complemented, and overexpressing strains of local regulators were inoculated into LB medium, with YD25 as control. Bacterial total RNA was extracted using the HiPure Bacterial RNA Kit (Magen), reverse-transcribed using TransScript First-Strand cDNA Synthesis SuperMix (Tiangen), and then real-time quantitative PCR was carried out using ChamQ SYBR qPCR Master Mix (Vazyme) to detect the expression level of *sdeY*, *sdeB*, *sdeQ*, *sdeC*, *and sdeG*, with r*poD* as the reference gene. Primer sequences are provided in Table S8.

The previous promoter reporter plasmids were electroporated into the knockout and overexpressing strains of global regulatory factors, respectively. β-galactosidase activity was determined using a modified Miller assay, following the same method as above.

### Determination of strains’ tolerance to xenobiotics impacted by regulatory factors

The knockout and overexpressing strains of global regulators were inoculated into LB medium and cultured overnight, with YD25 as a control. The strains were diluted with LB medium to an OD_600_ of 1.0, then serially diluted to 10^−10^. Starting from 10^−7^, 5 μL of each dilution culture was inoculated on LB solid plates containing 2% SDS, and the plates were incubated at 28°C for 16 h before recording the results.

On the other hand, the cultures of various strains with OD_600_ of 1.0 were inoculated at 1% into KB medium containing 1% bile salts and KB medium without bile salts, respectively. All cultures were incubated at 37°C until reaching an OD_600_ of 1.0. Crystal violet solution was added to the cultures (final concentration 5 μg/mL), and the reaction was proceeded at 37°C for 4 h. After the color of the cultures changing from blue to pink, 200 μL of 3% SDS solution was added to stop the reaction. The fluorescence was measured with an excitation wavelength of 560 nm and an emission wavelength of 590 nm. The experiment was repeated three times.

### Statistical analyses

Statistical analyses were performed using Microsoft Excel, and bar graphs or other statistical plots were generated using GraphPad Prism software. Data were obtained from at least three biological replicates. Details of the statistical tests, sample sizes, and methodologies are provided in the individual figure legends. Data are presented as mean ± SEM. One-way analysis of variance (ANOVA) or Student’s *t*-test was used to determine the statistical significance. “*” stands for *P* < 0.05, “**” for *P* < 0.01, “***” for *P* < 0.001, and “****” for *P* < 0.0001.

## Data Availability

All data from this study can be made available upon request, without limitation in time.

## SUPPLEMENTAL MATERIAL

Supplemental figures spectrum.02185-25-s0001.pdf Fig. S1 to S4.

Supplemental tables spectrum.02185-25-s0002.xlsx Tables S1 to S8.

ASM does not own the copyrights to Supplemental Material that may be linked to, or accessed through, an article. The authors have granted ASM a non-exclusive, world-wide license to publish the Supplemental Material files. Please contact the corresponding author directly for reuse.
