# Supplementary material for: Identification of oxyresveratrol, a food-as-medicine substrate, as a novel EPI that targets the RND efflux pumps of Serratia surfactantfaciens sp. nov. YD25T
Source: Microbiol Spectr. 2026 Apr 22;14(6):e02185-25. doi: 10.1128/spectrum.02185-25 (PMC13228086; doi:10.1128/spectrum.02185-25)
Supplement: Supplemental figures — Fig. S1 to S4. [file spectrum.02185-25-s0001.pdf]

Supplemental figure 1

A

	YD25
	MIC
CPZ	50 μg/m L
Oxyresveratrol	850 μg/m L
Evernic acid	85 μg/m L
Morin	340 μg/m L

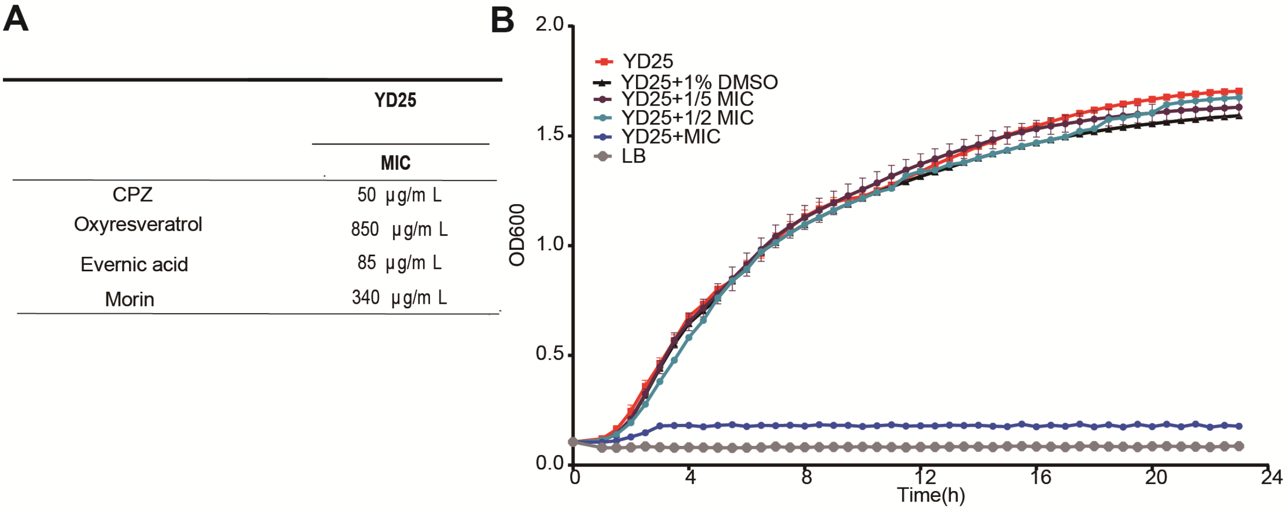

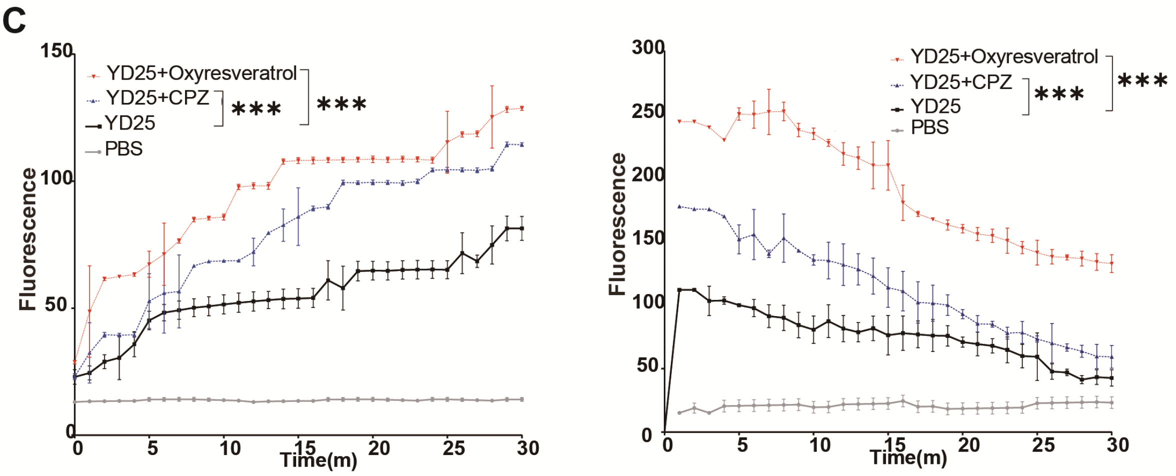

Figure S1. (A) MIC of some tested compounds against YD25. (B) Growth curve of YD25 treated with oxyresveratrol. The treatment concentrations of oxyresveratrol were MIC, 1/2 MIC, and 1/5 MIC, with 1% DMSO serving as the solvent control, alongside blank YD25 and LB medium controls. Hoechst accumulation and efflux assay for oxyresveratrol at 1/2×MIC (C). In each graph, the red line represents the compound-treated group, the blue line represents the CPZ-treated positive control group, the black line represents untreated YD25, and the gray line represents the blank PBS solution, with the x-axis denoting the treatment time and the y-axis denoting the fluorescence intensity.

Supplemental figure 2

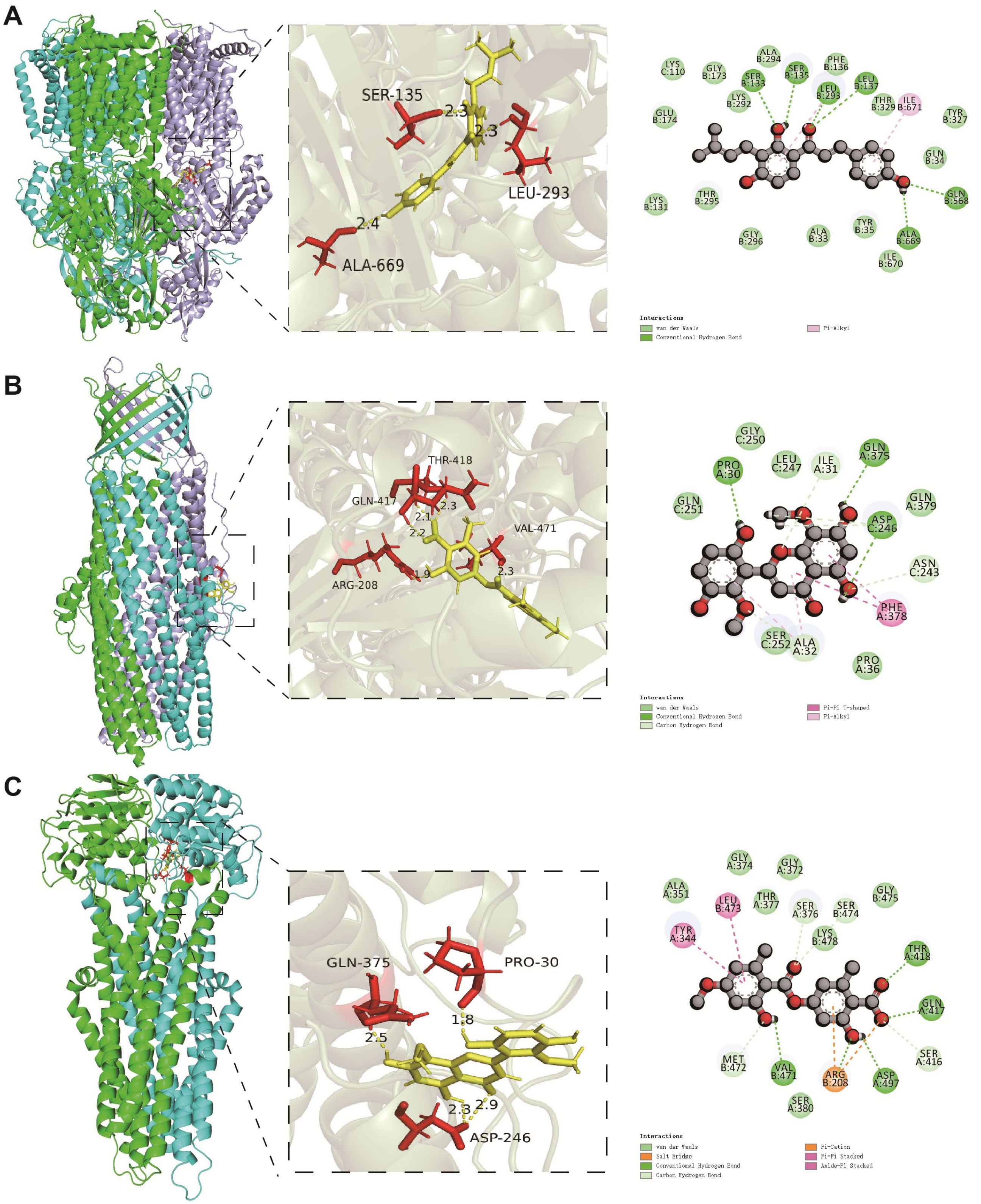

D

Interactions	SdeY protein binding with oxyresveratrol	SdeQ protein binding with morin	SmdB binding with evernic acid
Binding Energy (Kcal/mol)	-7.39	-8.25	-6.19
van der Waals	LYS110 GLU174 LYS131 GLY173 THR295 LYS292 GLY296 ALA294 ALA33 THR329 TYR35 ILE670 GLN34 TYR3327	GLN251 GLY250 LEU247 SER252 GLN379 PRO36	ALA351 GLY374 THR377 LYS478 GLY475 SER80
Pi-Alkel	ILE671	ALA32	
Conventional Hydrogen Bond	SER133 SER135 LEU293 ALA669 GLN568	PRO30 ASP246 GLN375	THR418 GLN417 ASP497 VAL471
Carbon Hydrogen Bond		ILE31 ALA32 ASN243	MET472 SER376 SER474 SER416
Pi-Pi T-shaped		PHE378	
Salt Eridge			ARG208
Amide-Pi Stacked			TYR344 LEU473

Figure S2. (A) Simulation diagram of the SdeY protein bound to oxyresveratrol. Left: In its native physiological state, SdeY forms a homotrimer, with individual monomers depicted in purple, green, and blue. The yellow stick model represents oxyresveratrol. Middle: Enlarged view of the binding pocket. Oxyresveratrol is shown as a yellow stick model, the gray translucent surface represents the electron density of the binding site, and black labels indicate key amino acid residues lining the pocket. Right: Two-dimensional interaction map illustrating the molecular contacts between SdeY and oxyresveratrol. (B) Simulation diagram of the SdeQ protein bound to morin. Left: In its native conformation, SdeQ exists as a homotrimer, with monomers colored purple, green, and blue. Morin is represented by a yellow stick model. Middle: Magnified view of the binding pocket. Morin is displayed as a blue stick model, the gray translucent surface outlines the electron cloud of the binding site, and black labels denote nearby amino acid residues. Right: Two-dimensional representation of the interactions between SdeQ and morin. (C) Simulation diagram of the SmdB protein bound to evernic acid. Left: Under physiological conditions, SmdB functions as a homodimer, with subunits shown in yellow and blue. Evernic acid is depicted as a pink stick model. Middle: Close-up view of the binding site. Evernic acid is illustrated as a green stick model, the gray translucent surface corresponds to the electron density of the binding pocket, and black annotations highlight adjacent amino acid residues. Right: Two-dimensional schematic of the intermolecular interactions between SmdB and evernic acid. (D) *In silico* estimated free binding energy and interactions of EPIs and efflux pump.

Supplemental figure 3

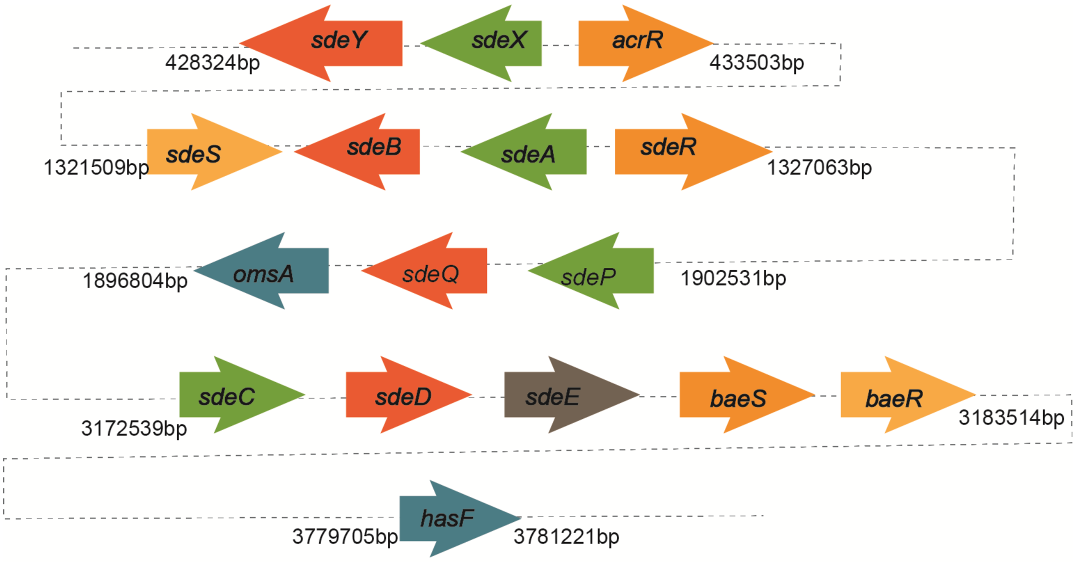

Figure S3. Schematic of the RND efflux pump genes and their local regulator genes in YD25. Red for RND protein, green for the membrane fusion protein, blue for the outer membrane protein, and yellow for the local regulator.

Supplemental figure 4

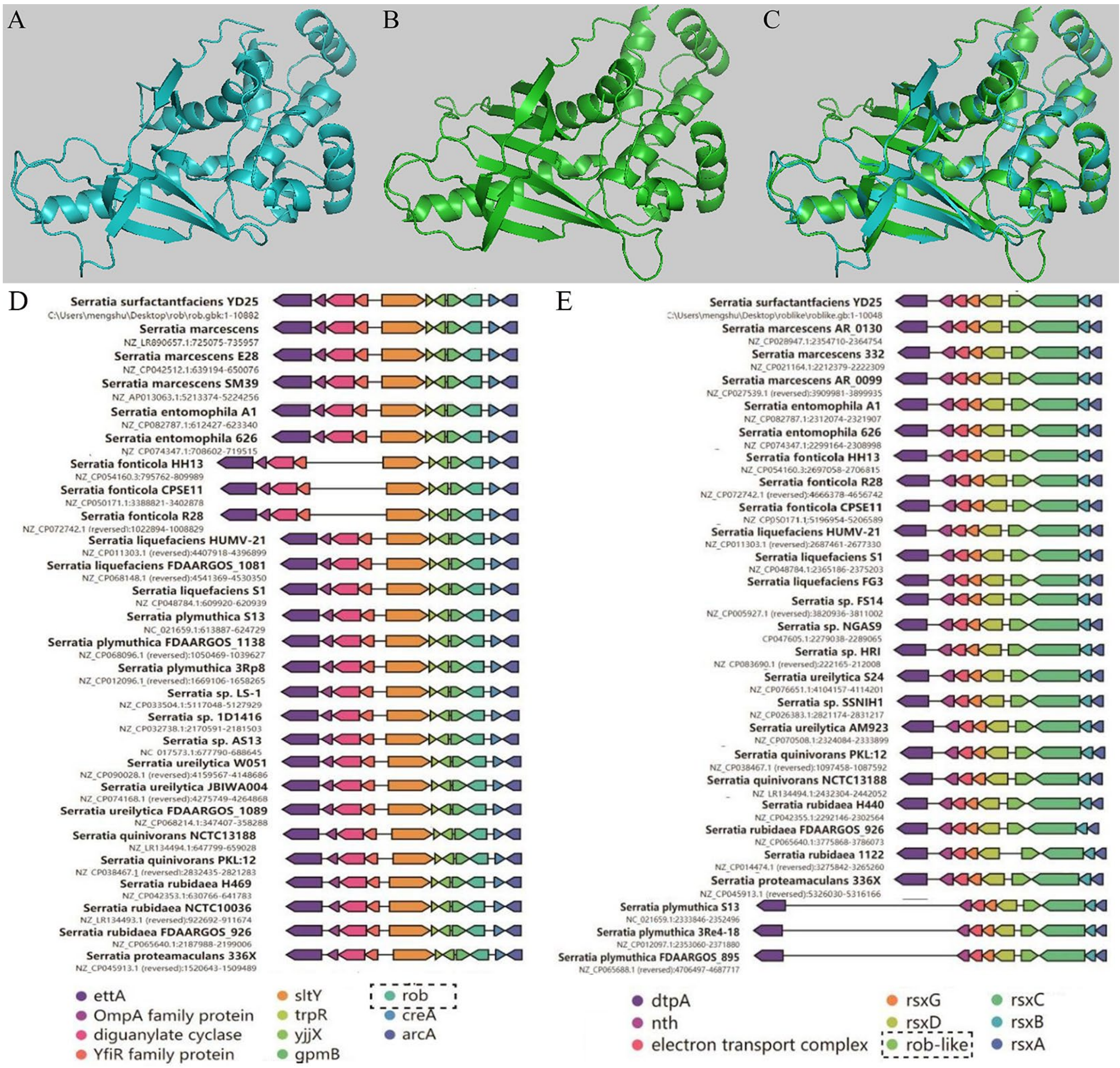

Figure S4. Protein tertiary structure simulation diagrams of Rob protein (A), Roblike protein (B), and the overlay of Rob and Roblike (C). Genomic loci of *rob* (D) and *roblike* (E) in 161 *Serratia* genomes (partial results).
